# Statistics of Insanity
*Statistics of Insanity; being a Decennial Report of Bethlem Hospital, from 1846 to 1855, inclusive. By W. Charles Hood, M.D. London: Batten. 1857.


**Published:** 1857-07

**Authors:** 


### STATISTICS OF INSANITY.*

These statistics of insanity contain much interesting matter relative to the causes of insanity, and to recoveries and deaths. Dr. Hood bears strong testimony in favour of active exercise as an incentive to mental restoration, this being the only hygienic subject introduced.

"At a certain period of convalescence," he observes, "long walks beyond the precincts of the Asylum, under proper surveillance, are of the highest utility, as has been abundantly proved in the experience of Bethlem and elsewhere. Amusements, such as cricket, bowls, or skittles, are also of great use; and so are all the amusements which can be commanded within-doors. But occupation is even of greater importance than amusement; indeed systematic employment, in various forms, is now a duly recognized essential to successful treatment in all properly conducted Asylums. Pinel recommended that an asylum should have a farm connected with it, in which patients could labour; and great credit is due to the late Sir William Ellis for having called attention to this principle, in this country, by giving appropriate work to the patients successively under his care in Wakefield and Hanwell. Esquirol mentions a fact from Bourgoise's Travels in Spain which speaks volumes on this subject: it is, 'that the rich in the Hospital for the Insane at Saragossa are not restored in the same ratio as the poor, *because* they are not obliged to labour.'"

* Statistics of Insanity; being a Decennial Report of Bethlem Hospital, from 1846 to 1855, inclusive. By W. Charles Hood, M.D. London: Batten. 1857.

